# Settlement cue selectivity by larvae of the destructive crown-of-thorns starfish

**DOI:** 10.1098/rsbl.2022.0399

**Published:** 2023-01-25

**Authors:** Peter C. Doll, Sven Uthicke, Ciemon F. Caballes, Guillermo Diaz-Pulido, Muhammad A. Abdul Wahab, Bethan J. Lang, So Young Jeong, Morgan S. Pratchett

**Affiliations:** ^1^ Australian Research Council (ARC) Centre of Excellence for Coral Reef Studies, James Cook University, Townsville, Queensland 4811, Australia; ^2^ Australian Institute of Marine Science, Townsville, Queensland 4810, Australia; ^3^ National Science Foundation Established Program to Stimulate Competitive Research (NSF EPSCoR) - Guam Ecosystems Collaboratorium for Corals and Oceans, University of Guam – Marine Laboratory, Mangilao, Guam 96923, USA; ^4^ School of Environment and Science, Coastal and Marine Research Centre, and Australian Rivers Institute, Griffith University, Nathan, Queensland 4111, Australia

**Keywords:** coralline algae, *Acanthaster*, population outbreaks, larval settlement, metamorphosis, recruitment

## Abstract

Population irruptions of crown-of-thorns starfish (COTS) cause extensive degradation of coral reefs, threatening the structure and function of these important ecosystems. For population irruptions to initiate and spread, large numbers of planktonic larvae have to successfully transition into their benthic life-history stage (i.e. settlement), whereby larval behaviour and the presence of settlement cues may shape spatial patterns of recruitment and adult densities. Our results demonstrate that a wide range of coralline algae species induce COTS larvae to settle; however, the capacity to promote settlement success varied manyfold among algal species, ranging from greater than 90% in *Melyvonnea* cf. *madagascariensis* to less than 2% in *Lithophyllum* cf. *kotschyanum* and two *Porolithon* species at 24 h. Because many coralline algae species that promote high settlement success are prevalent in shallow reef habitats, our findings challenge the hypothesis that COTS larvae predominantly settle in deep water. Considering both larval behaviour and algal ecology, this study highlights the ecological significance of coralline algae communities in driving recruitment patterns of COTS. More specifically, the local abundance of highly inductive coralline algae (especially, *Melyvonnea* cf. *madagascariensis*) may explain some of the marked spatial heterogeneity of COTS populations and the incidence of population irruptions.

## 1. Introduction

Environmental cues play pivotal roles in the regulation of animal life histories, particularly in the timing and completion of major life-history transitions [1,2]. Response mechanisms to environmental cues permit animals to orchestrate these transitions and optimize survival at transition points which are characterized by high mortality [3,4]. For many animals with complex life cycles, survival rates are particularly low at ontogenetic boundaries between early life-history stages [3,5,6]. Consequently, the presence of apt environmental cues, combined with the capability of animals to respond to them during early ontogeny, can drastically change the dynamics of populations [7–9].

The transition from larval to juvenile stages, which in most animals includes some form of metamorphosis [10–12], is largely governed by environmental cues [13]. Groups of insects, fishes, amphibians and marine invertebrates have evolved neural and hormonal mechanisms translating abiotic and biotic cues to navigate this inherently vulnerable phase [14–16]. Most benthic marine invertebrates display bipartite life histories with a highly specialized larval settlement stage [17]. This irreversible planktonic–benthic transition (i.e. settlement) can be a major population bottleneck [18,19]. However, response to environmental inputs (e.g. chemical cues associated with conspecifics or benthic substrata) may result in the settlement of larvae in locations that confer higher likelihood of survival and recruitment [20–22]. The larval decision of where and when to settle is thus of fundamental importance.

Population irruptions of crown-of-thorns starfish (*Acanthaster* spp., COTS) remain a significant driver of coral loss and reef degradation [23], which are increasingly compounded by climatic disturbance [24,25]. For population irruptions to occur and spread among coral reefs, large numbers of planktonic larvae must successfully transition into the benthic juvenile stage [26,27]. In contrast with other echinoderm larvae that metamorphose during their planktonic stage [22], COTS metamorphosis is initiated after substratum contact [26]. Because of exceptionally high mortality rates in early juvenile COTS [28,29] and limited adult movement behaviour [30,31], settlement rates are likely to be the foremost constraint on local abundance and the incidence of population irruptions [32–34]. Larval settlement of COTS in the wild is presumably induced by coralline algae and their associated microbial communities [35]; however, it is unclear whether all or only some coralline algae have the capacity to induce high settlement rates [23]. The alga *Lithothamnion* cf. *proliferum* was so far surmised to be the predominant settlement cue, which gave rise to the hypothesis that COTS mostly settle in deep, inter-reef habitats [36]. Recent advances in the taxonomy and contrasting ecology of different coralline algae species do, however, necessitate a renewed exploration of settlement induction.

Here, we assess the relative capacity of a diversity of coralline algae to induce COTS settlement in order to test the hypothesis that the alga *Lithothamnion* cf. *proliferum* promotes higher settlement rates than the other species. Notably, this research facilitates a critical evaluation of the deep-water recruitment hypothesis [36] by considering whether other algal species that play important roles in COTS settlement induction occur in deep and/or shallow reef habitats. The integration of larger-scale algal field-distribution data in the interpretation of our experimental results further enables us to better understand the ecological consequences of coralline algae assemblages for the recruitment patterns and ecological impact of this nuisance starfish.

## 2. Materials and methods

To obtain settlement-stage western Pacific COTS (*Acanthaster* cf. *solaris*) for experimental assays, we reared larvae at the Australian Institute of Marine Science (AIMS) National Sea Simulator (electronic supplementary material, §S1), following Uthicke *et al*. [37]. Larval development was microscopically examined until we determined metamorphic competency 14 days post-fertilization. Experimental treatments included 14 living coralline algae species and one living Peyssonneliaceae alga (collectively referred to as coralline algae in this study; table 1) with relatively high abundance on Australia's Great Barrier Reef (GBR), and structural control (sterile aragonite) and filtered seawater (FSW) control treatments. A diversity of coralline algae were collected from two central GBR locations (electronic supplementary material, §S2), identified based on morpho-anatomical features (electronic supplementary material, §S2) and molecular sequencing (electronic supplementary material, §S3, §S4, following [40]), and cut into replicate 5 × 5 mm live chips for use in experiments.

**Table 1.** Ecological information on the 15 coralline algae species analysed in settlement assays. Relative abundance along the GBR shelf is categorized as rare (less than 20%), moderate (20–70%) and common (greater than 70%), largely calculated based on total abundance data reported in Dean *et al*. [38] (e.g. species abundance in ‘outer’ reefs divided by the species abundance across all three shelf positions). Taxonomic, morpho-anatomical and collection information are provided in the electronic supplementary material (§S2).

species	habitat	irradiance level	relative abundance (GBR shelf)			source (GBR abundance)
			inner	mid	outer	
*Melyvonnea* cf. *madagascariensis*	shallow - deep reef	low - mid	rare	common	rare	[38]
*Neogoniolithon fosliei*	crest, shallow reef	high	rare	rare	common	[38]
*Adeylithon bosencei*	shallow - deep reef	low - high	rare	rare	common	[39], G.D.-P. pers. obs.
*Hydrolithon* cf. *reinboldii*	shallow - deep reef	mid	moderate	rare	moderate	[38]
*Lithophyllum* cf. *insipidum*	crest, shallow reef	mid - high	rare	moderate	moderate	[38]
*Lithothamnion* cf. *proliferum*	crevices, caves	low	rare	common	rare	[38]
*Titanoderma* cf. *tessellatum*	shallow - deep reef	low - mid	rare	moderate	moderate	[38]
*Amphiroa foliacea*	shallow - mid reef	mid - high	rare	moderate	moderate	G.D.-P. pers. obs.
*Sporolithon* sp.	crevices, caves	low	rare	rare	common	[38]
*Ramicrusta* sp.	crevices, caves	low	rare	moderate	moderate	G.D.-P. pers. obs.
*Lithophyllum* cf. *pygmaeum*	crest, shallow reef	mid - high	rare	moderate	moderate	[38]
*Porolithon* sp. A	reef crest	high	common	rare	rare	G.D.-P. pers. obs.
*Porolithon* sp. B	reef crest	high	rare	moderate	moderate	[38]
*Lithophyllum* cf. *kotschyanum*	reef crest	mid - high	rare	moderate	moderate	[38]
*Porolithon* sp. C	reef crest	high	rare	moderate	moderate	[38]

**Figure 1.**
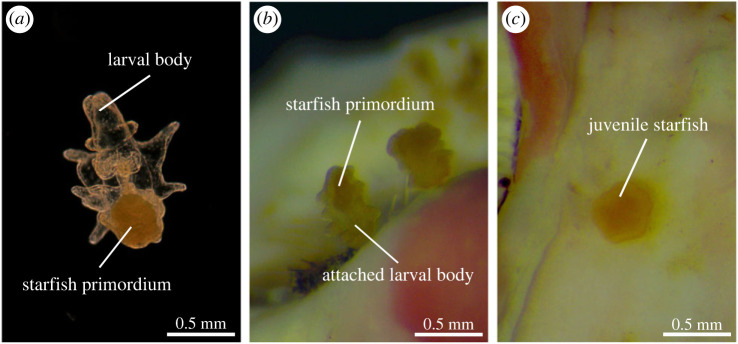
Larval development through metamorphosis in crown-of-thorns starfish: (*a*) brachiolaria larva; (*b*) metamorphosing larva absorbing the larval body; (*c*) post-metamorphic juvenile (photographs by Ciemon Caballes and Peter Doll).

To test the effects of different coralline algae species on the settlement response of competent COTS larvae, 12 replicate settlement assays were conducted for each of the 17 experimental treatments. We used six-well cell culture plates and fully randomized the distribution of all replicate assays among the 204 wells. After adding 10 ml FSW and a single chip of one of 15 different algal species or sterile aragonite to the wells, we carefully introduced approximately 10 competent COTS larvae per well using glass pipettes. All well plates were kept in a temperature-controlled room (28°C) matching the light conditions during larval rearing (12 L : 12 D, light–dark). Using stereo microscopes, larval settlement was scored 24 and 48 h after larvae were introduced. For each replicate well, we recorded the number of competent late-brachiolaria larvae remaining in the water column (= swimming) and the number of individuals that had successfully attached to the treatment chip or well bottom and commenced or completed metamorphic transformation into a juvenile with radial symmetry (= settled, figure 1).

Statistical analyses were performed using R software (v. 4.1.3. [41]). To compare the responses of COTS larvae to coralline algae and control treatments, we considered the proportion of settled postlarvae and swimming larvae in each assay well. The effects of treatments and time (24, 48 h) on this proportional data frame were modelled using a binomial generalized linear model with a logit link (*stats* package [41]). This model was fitted with the bias-reduction method *brglmFit* [42,43] from the *brglm2* package [44] to avoid data separation due to outcomes with only zeros in the control treatments. Model assumptions were evaluated based on inspection of diagnostic plots and figures were generated using the *ggplot2* package [45]. We calculated estimated marginal means, confidence intervals and post hoc comparisons using the *emmeans* package [46] and the *cld* function from the *multcomp* package [47]. To account for multiple comparisons and control for the false discovery rate, *p*-values (alpha = 0.05) were adjusted using the *BY* correction method following the Benjamini–Yekutieli procedure [48].

## 3. Results

**Figure 2.**
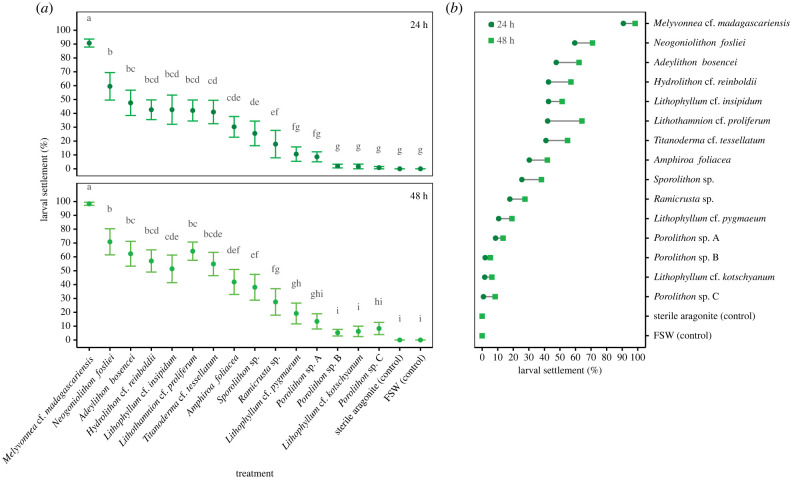
(*a*) Settlement cue responses of crown-of-thorns starfish larvae to 15 coralline algae species at 24 and 48 h (means ± s.e., *n* = 12). Letters denote statistical differences among treatments, with treatment means not sharing any letter being significantly different (Benjamini–Yekutieli-adjusted *p* < 0.05). (*b*) Differences between the mean settlement rates at 24 and 48 h after experiment commencement. FSW = filtered seawater.

While larval settlement was induced in all coralline algae treatments (table 1, figure 2*a*), no larvae settled in both controls and settlement success differed substantially among the 17 treatments (figure 2*a*, *F*_16,391_ = 7.97, *p* < 0.001). Highest settlement rates (mean ± s.e.: 90.7% ± 2.9 at 24 h; 98.3% ± 1.1 at 48 h) were recorded in the presence of *Melyvonnea* cf. *madagascariensis*, while limited settlement was recorded (less than 2% at 24 h; <10% at 48 h) for two *Porolithon* species and *Lithophyllum* cf. *kotschyanum*. Settlement rates were relatively high (30–60% at 24 h) for seven coralline algae treatments, with an evident hierarchy in larval settlement responses to cues associated with different coralline species (figure 2*a*). Settlement rates differed significantly between scoring times (*F*_1,390_ = 62.97, *p* < 0.001), although there was no interaction with treatments (figure 2*b*, *F*_16,374_ = 0.52, *p* = 0.939), reflecting consistent differences in settlement rates among different algal species.

## 4. Discussion

Settlement cues and inducers are critically important in regulating the abundance of many different benthic marine invertebrates [21], yet there is very limited understanding of the factors that influence settlement rates of COTS [23]. Our results demonstrate that a wide range of crustose and articulated (geniculate) coralline algae induce COTS settlement, though there were marked differences in settlement rates associated with different algal species. Although it remains to be resolved whether such differences are driven by algal compounds and/or associated microbial communities (but see [35]), this study corroborates the role of chemical cues in governing benthic habitat selection by planktonic COTS larvae. Similar to other echinoderm groups [22], COTS larvae display active searching and testing behaviour [49,50] to detect chemical cues that presumably signal the presence of preferred early-juvenile food (i.e. coralline algae [51,52]) or the absence of toxic surfaces and coral polyps that can injure juveniles [53]. Physical microhabitat characteristics (electronic supplementary material, §S2) and the irradiance conditions that algal species occur in (table 1) do not appear to be primary factors in COTS settlement induction; however, in the presence of a suitable settlement cue, larvae likely select low-light microhabitats within the reef matrix for shelter during metamorphosis [54].

Importantly, our results indicate that COTS larvae do not require cues associated with the coralline alga *Lithothamnion* cf. *proliferum* or other deep-water species for settlement induction. Many algal species that are prevalent at moderate and shallow depths (table 1) induced high rates of settlement in this study, including species that primarily occur in shallow reef environments (e.g. *Neogoniolithon fosliei* and *Adeylithon bosencei*). Most notably, the alga *Melyvonnea* cf. *madagascariensis*, which is widely distributed across the depth continuum, promoted considerably higher settlement success than *Lithothamnion* cf. *proliferum* (likely *Lithothamnium pseudosorum* in [36]). In part because the latter species was previously considered to be the gold standard for COTS settlement induction [35,36] and assumed to be relatively rare at moderate and shallow depths [36], the deep-water recruitment hypothesis proposed that COTS larvae predominantly settle in deep (greater than 30 m), inter-reef habitats [36]. However, this species can be abundant in crevices, caves and overhangs in shallow water (G.D.-P., pers. obs.). Our findings further challenge this hypothesis by demonstrating a shallow-water prevalence of suitable settlement cues, which is supported by recorded settlement [34] and newly settled juveniles [55] at a range of shallow to intermediate depths (1–18 m).

Apparent selectivity for particular coralline algae by settling COTS larvae has potential importance for understanding population dynamics, because settlement delay and settlement in adverse environments can negatively impact recruitment success [17,56]. Marine invertebrate larvae with a specialized settlement stage generally respond to environmental cues to maximize recruitment rates [20], but limited plasticity in settlement behaviour and narrow habitat requirements imply dependence on encountering suitable habitat at small spatio-temporal scales. There is laboratory-based evidence that COTS larvae can extend their competency periods in the absence of environmental cues [57], and our results show that some larvae temporarily defer settlement even in the presence of settlement cues. However, delayed settlement will increase exposure to predators [58,59] and can reduce growth and subsequent survival in early juveniles [17,56]. Specificity in substratum selection may decrease during the competency period and larvae are more likely to eventually settle in adverse environments [60], increasing susceptibility of juveniles to benthic predation [33,61] and starvation [33,62]. Elevated mortality rates before and after settlement may consequently limit recruitment success in habitats with limited settlement cues.

More specifically, the availability of suitable settlement habitat may play a critical role in explaining inter-reef differences in the abundance of COTS and the incidence of population irruptions. Our findings indicate that specific algal species (e.g. *Melyvonnea* cf. *madagascariensis*) may be particularly important for promoting or regulating natural settlement rates of COTS. Population irruptions degrading the GBR predominantly occur on reefs in the mid-section of the continental shelf [63], raising questions pertaining to potential drivers of low adult densities on inshore reefs [23]. Limited inter-reef connectivity may constrain larval supply in some locations, yet many inshore reefs have been highlighted as significant larval sinks [64], pre-disposing them to COTS infestations. Food limitation of planktotrophic larvae is also unlikely to disproportionately inhibit recruitment on inshore reefs given the relatively high concentrations of large phytoplankton in these locations [65]. Instead, the low overall abundance of coralline substrata on inshore reefs [38,66,67] likely contributes to reduced settlement success in these locations. Moreover, the coralline algae species inducing relatively high settlement rates in this study, except *Hydrolithon* cf. *reinboldii*, are absent or relatively rare in most inshore reefs (table 1, [38,39,68]. We hypothesize, therefore, that low abundance of coralline algae, particularly of species promoting high settlement rates, poses a significant constraint for COTS recruitment on many inshore reefs. However, *in situ* studies of settlement rates and coralline algae assemblages across this shelf gradient are required to substantiate this hypothesis. Notably, the combination of limited larval supply and scarcity of suitable settlement habitat would greatly reduce localized risk of COTS population irruptions and associated coral loss.

In the face of ever-increasing threats to coral reefs, it appears essential to consider all mechanisms that determine recruitment success in this keystone coral predator, both from a theoretical (e.g. life-history theory) and applied perspective (e.g. population control). While previously overlooked in the discussion of factors contributing to the incidence of COTS population irruptions, larval settlement behaviour and settlement cue availability emerge as factors capable of explaining some of the marked spatial heterogeneity in recruitment and adult population sizes.

## Data Availability

The molecular sequences for each algal species can be accessed in the GenBank database under accession numbers OP830444 to OP830473 (electronic supplementary material, §S4, [69]), and the experimental assay data and description are available from the Dryad Digital Repository: https://doi.org/10.5061/dryad.gqnk98srv [70]. Data are provided in the electronic supplementary material [71].

## References

[1] Shipley JR, Twining CW, Taff CC, Vitousek MN, Flack A, Winkler DW. 2020 Birds advancing lay dates with warming springs face greater risk of chick mortality. Proc. Natl Acad. Sci. USA **117**, 25 590-25 594. (10.1073/pnas.2009864117) PMC7568286 32989166

[2] Oestreich WK, Abrahms B, McKenna M, Goldbogen JA, Crowder LB, Ryan JP. 2022 Acoustic signature reveals blue whales tune life-history transitions to oceanographic conditions. Funct. Ecol. **36**, 882-895. (10.1111/1365-2435.14013)

[3] Low M, Pärt T. 2009 Patterns of mortality for each life-history stage in a population of the endangered New Zealand stitchbird. J. Anim. Ecol. **78**, 761-771. (10.1111/j.1365-2656.2009.01543.x) 19302320

[4] Visser ME, Gienapp P. 2019 Evolutionary and demographic consequences of phenological mismatches. Nat. Ecol. Evol. **3**, 879-885. (10.1038/s41559-019-0880-8) 31011176 PMC6544530

[5] Sullivan KA. 1989 Predation and starvation: age-specific mortality in juvenile juncos (*Junco phaenotus*). J. Anim. Ecol. **58**, 275-286. (10.2307/5000)

[6] Gaillard JM, Festa-Bianchet M, Yoccoz NG. 1998 Population dynamics of large herbivores: variable recruitment with constant adult survival. Trends Ecol. Evol. **13**, 58-63. (10.1016/S0169-5347(97)01237-8) 21238201

[7] Gaillard JM, Boutin JM, Delorme D, Van Laere G, Duncan P, Lebreton JD. 1997 Early survival in roe deer: causes and consequences of cohort variation in two contrasted populations. Oecologia **112**, 502-513. (10.1007/s004420050338) 28307627

[8] Sæther BE, Tufto J, Engen S, Jerstad K, Røstad OW, Skåtan JE. 2000 Population dynamical consequences of climate change for a small temperate songbird. Science **287**, 854-856. (10.1126/science.287.5454.854) 10657299

[9] Ozgul A, Childs DZ, Oli MK, Armitage KB, Blumstein DT, Olson LE, Tuljapurkar S, Coulson T. 2010 Coupled dynamics of body mass and population growth in response to environmental change. Nature **466**, 482-485. (10.1038/nature09210) 20651690 PMC5677226

[10] Truman JW, Riddiford LM. 1999 The origins of insect metamorphosis. Nature **401**, 447-452. (10.1038/46737) 10519548

[11] Suzuki Y, Koyama T, Hiruma K, Riddliford LM, Truman JW. 2013 A molt timer is involved in the metamorphic molt in *Manduca sexta* larvae. Proc. Natl Acad. Sci. USA **110**, 12 518-12 525. (10.1073/pnas.1311405110) PMC3732944 23852731

[12] Nagamine K, Ishikawa Y, Hoshizaka S. 2016 Insights into how longicorn beetle larvae determine the timing of metamorphosis: starvation-induced mechanism revisited. PLoS ONE **11**, e0158831. 27386861 10.1371/journal.pone.0158831 PMC4936689

[13] Laudet V. 2011 The origins and evolution of vertebrate metamorphosis. Curr. Biol. **21**, 726-737. (10.1016/j.cub.2011.07.030) 21959163

[14] Denver RJ. 1997 Environmental stress as a developmental cue: corticotropin-releasing hormone is a proximate mediator of adaptive phenotypic plasticity in amphibian metamorphosis. Horm. Behav. **31**, 169-179. (10.1006/hbeh.1997.1383) 9154437

[15] Doherty PJ, Dufour V, Galzin R, Hixon MA, Meekan MG, Planes S. 2004 High mortality during settlement is a population bottleneck for a tropical surgeonfish. Ecology **85**, 2422-2428. (10.1890/04-0366)

[16] Lowe WH, Martin TE, Skelly DK, Woods HA. 2021 Metamorphosis in an era of increasing climate variability. Trends Ecol. Evol. **36**, 360-375. (10.1016/j.tree.2020.11.012) 33414021

[17] Rodríguez SR, Ojeda FP, Inestrosa NC. 1993 Settlement of benthic marine invertebrates. Mar. Ecol. Prog. Ser. **97**, 193-207. (10.3354/meps097193)

[18] Underwood AJ, Fairweather PG. 1989 Supply-side ecology and benthic marine assemblages. Trends Ecol. Evol. **4**, 16-20. (10.1016/0169-5347(89)90008-6) 21227303

[19] Hunt HL, Scheibling RE. 1997 Role of early post-settlement mortality in recruitment of benthic marine invertebrates. Mar. Ecol. Prog. Ser. **155**, 269-301. (10.3354/meps155269)

[20] Pawlik JR. 1992 Chemical ecology of the settlement of benthic marine invertebrates. Oceanogr. Mar. Biol. **30**, 273-335.

[21] Hadfield MG, Paul VJ. 2001 Natural chemical cues for settlement and metamorphosis of marine invertebrate larvae. In Marine chemical ecology (eds J McClintock, B Baker), pp. 431-461. Boca Raton, FL: CRC Press.

[22] Doll PC, Caballes CF, Hoey AS, Uthicke S, Ling SD, Pratchett MS. 2022 Larval settlement in echinoderms: a review of processes and patterns. Oceanogr. Mar. Biol. **60**, 433-494.

[23] Pratchett MS et al. 2021 Knowledge gaps in the biology, ecology, and management of the Pacific crown-of-thorns sea star, *Acanthaster* sp., on Australia's Great Barrier Reef. Biol. Bull. **241**, 330-346. (10.1086/717026) 35015620

[24] Mellin C et al. 2019 Spatial resilience of the Great Barrier Reef under cumulative disturbance impacts. Glob. Chang. Biol. **25**, 2431-2445. (10.1111/gcb.14625) 30900790

[25] Castro-Sanguino C et al. 2021 Reef state and performance as indicators of cumulative impacts on coral reefs. Ecol. Indic. **123**, 107335. (10.1016/j.ecolind.2020.107335)

[26] Pratchett MS, Caballes CF, Rivera-Posada JA, Sweatman HPA. 2014 Limits to understanding and managing outbreaks of crown-of-thorns starfish (*Acanthaster* spp.). Oceanogr. Mar. Biol. **52**, 133-200. (10.1201/b17143-4)

[27] Deaker DJ, Byrne M. 2022 Crown of thorns starfish life-history traits contribute to outbreaks, a continuing concern for coral reefs. Emerg. Top. Life Sci. **6**, 67-79. (10.1042/ETLS20210239) 35225331 PMC9023020

[28] Keesing JK, Halford AR. 1992 Importance of post-settlement processes for the population dynamics of *Acanthaster planci* (L.). Mar. Freshw. Res. **43**, 635-651. (10.1071/MF9920635)

[29] Keesing JK, Halford AR, Hall KC. 2018 Mortality rates of small juvenile crown-of-thorns starfish *Acanthaster planci* on the Great Barrier Reef: implications for population size and larval settlement thresholds for outbreaks. Mar. Ecol. Prog. Ser. **597**, 179-190. (10.3354/meps12606)

[30] Pratchett MS, Cowan ZL, Nadler LE, Caballes CF, Hoey AS, Messmer V, Fletcher CS, Westcott DA, Ling SD. 2017 Body size and substrate type modulate movement by the western Pacific crown-of-thorns starfish, *Acanthaster solaris*. PloS ONE **12**, e0180805. (10.1371/journal.pone.0180805) 28877193 PMC5587101

[31] Ling SD, Cowan ZL, Boada J, Flukes EB, Pratchett MS. 2020 Homing behaviour by destructive crown-of-thorns starfish is triggered by local availability of coral prey. Proc. R. Soc. B **287**, 20201341. (10.1098/rspb.2020.1341) PMC7735281 33143585

[32] MacNeil MA, Chong-Seng KM, Pratchett DJ, Thompson CA, Messmer V, Pratchett MS. 2017 Age and growth of an outbreaking *Acanthaster* cf. *solaris* population within the Great Barrier Reef. Diversity **9**, 18. (10.3390/d9010018)

[33] Wilmes JC, Caballes CF, Cowan ZL, Hoey AS, Lang BJ, Messmer V, Pratchett MS. 2018 Contributions of pre-versus post-settlement processes to fluctuating abundance of crown-of-thorns starfishes (*Acanthaster* spp.). Mar. Poll. Bull. **135**, 332-345. (10.1016/j.marpolbul.2018.07.006) 30301045

[34] Doll PC, Messmer V, Uthicke S, Doyle JR, Caballes CF, Pratchett MS. 2021 DNA-based detection and patterns of larval settlement of the corallivorous crown-of-thorns sea star (*Acanthaster* sp.). Biol. Bull. **241**, 271-285. (10.1086/717539) 35015627

[35] Johnson CR, Sutton DC. 1994 Bacteria on the surface of crustose coralline algae induce metamorphosis of the crown-of-thorns starfish *Acanthaster planci*. Mar. Biol. **120**, 305-310. (10.1007/BF00349692)

[36] Johnson CR, Sutton DC, Olson RR, Giddins R. 1991 Settlement of crown-of-thorns starfish: role of bacteria on surfaces of coralline algae and a hypothesis for deep-water recruitment. Mar. Ecol. Prog. Ser. **71**, 143-162. (10.3354/meps071143)

[37] Uthicke S, Logan M, Liddy M, Francis D, Hardy N, Lamare M. 2015 Climate change as an unexpected co-factor promoting coral eating seastar (*Acanthaster planci*) outbreaks. Sci. Rep. **5**, 8402. (10.1038/srep08402) 25672480 PMC4325318

[38] Dean AJ, Steneck RS, Tager D, Pandolfi JM. 2015 Distribution, abundance and diversity of crustose coralline algae on the Great Barrier Reef. Coral Reefs **34**, 581-594. (10.1007/s00338-015-1263-5)

[39] Steneck RS. 1982 Adaptive trends in the ecology and evolution of crustose coralline algae (Rhodophyta, Corallinaceae). PhD thesis, Johns Hopkins University, Baltimore, MD.

[40] Jeong SY, Diaz-Pulido G, Maneveldt GW, Gabrielson PW, Nelson WA, Won BY, Cho TO. 2022 *Phymatolithopsis* gen. nov. (Hapalidiales, Corallinophycidae, Rhodophyta) based on molecular and morpho-anatomical evidence. J. Phycol. **58**, 161-178. (10.1111/jpy.13227) 34862980

[41] R Core Team. 2022 R: a language and environment for statistical computing. Vienna, Austria: R Foundation for Statistical Computing.

[42] Firth D. 1993 Bias reduction of maximum likelihood estimates. Biometrika **80**, 27-38. (10.1093/biomet/80.1.27)

[43] Kosmidis I, Firth D. 2009 A generic algorithm for reducing bias in parametric estimation. Electron. J. Stat. **4**, 1097-1112.

[44] Kosmidis I, Kenne Pagui EC, Sartori N. 2020 Mean and median bias reduction in generalized linear models. Stat. Comput. **30**, 43-59. (10.1007/s11222-019-09860-6)

[45] Wickham H. 2016 Ggplot2: elegant graphics for data analysis. New York, NY: Springer-Verlag.

[46] Lenth RV. 2022 emmeans: Estimated Marginal Means, aka Least-Squares Means. R package version 1.7.3. See https://cran.r-project.org/web/packages/emmeans/index.html.

[47] Hothorn T, Bretz F, Westfall P. 2008 Simultaneous inference in general parametric models. Biom. J. **50**, 346-363. (10.1002/bimj.200810425) 18481363

[48] Benjamini Y, Yekutieli D. 2001 The control of false discovery rate in multiple testing under dependency. Ann. Statist. **29**, 1165-1188. (10.1214/aos/1013699998)

[49] Henderson JA, Lucas JS. 1971 Larval development and metamorphosis of *Acanthaster planci* (Asteroidea). Nature **232**, 655-657. (10.1038/232655a0) 16063148

[50] Yamaguchi M. 1973 Early life histories of coral reef asteroids, with special reference to *Acanthaster planci* (L.). In Biology and geology of coral reefs (Vol. 2: biology) (eds OA Jones, R Endean), pp. 369-387. New York, NY: Academic Press.

[51] Deaker DJ, Agüera A, Lin HA, Lawson C, Budden C, Dworjanyn SA, Mos B, Byrne M. 2020 The hidden army: corallivorous crown-of-thorns seastars can spend years as herbivorous juveniles. Biol. Lett. **16**, 20190849. (10.1098/rsbl.2019.0849) 32264781 PMC7211459

[52] Wilmes JC, Hoey AS, Pratchett MS. 2020 Contrasting size and fate of juvenile crown-of-thorns starfish linked to ontogenetic diet shifts. Proc. R. Soc. B **287**, 20201052. (10.1098/rspb.2020.1052) PMC7423671 32693724

[53] Chesher RH. 1969 Destruction of Pacific corals by the sea star *Acanthaster planci*. Science **165**, 280-283. (10.1126/science.165.3890.280) 17814827

[54] Caballes CF, Pratchett MS. 2014 Reproductive biology and early life history of the crown-of-thorns starfish. In Echinoderms: ecology, habitats and reproductive biology (ed. E Whitmore), pp. 101-146. New York, NY: Nova Science Publishers.

[55] Wilmes JC, Schultz DJ, Hoey AS, Messmer V, Pratchett MS. 2020 Habitat associations of settlement-stage crown-of-thorns starfish on Australia's Great Barrier Reef. Coral Reefs **39**, 1163-1174. (10.1007/s00338-020-01950-6)

[56] Pechenik J. 1990 Delayed metamorphosis by larvae of benthic marine-invertebrates—does it occur? Is there a price to pay? Ophelia **32**, 63-94. (10.1080/00785236.1990.10422025)

[57] Pratchett MS, Dworjanyn SA, Mos B, Caballes CF, Thompson CA, Blowes S. 2017 Larval survivorship and settlement of crown-of-thorns starfish (*Acanthaster* cf. *solaris*) at varying algal cell densities. Diversity **9**, 2. (10.3390/d9010002)

[58] Cowan ZL, Pratchett M, Messmer V, Ling S. 2017 Known predators of crown-of-thorns starfish (*Acanthaster* spp.) and their role in mitigating, if not preventing, population outbreaks. Diversity **9**, 7. (10.3390/d9010007)

[59] Cowan ZL, Ling SD, Caballes CF, Dworjanyn SA, Pratchett MS. 2020 Crown-of-thorns starfish larvae are vulnerable to predation even in the presence of alternative prey. Coral Reefs **29**, 293-303. (10.1007/s00338-019-01890-w)

[60] Meyer KS, Wheeler JD, Houlihan E, Mullineaux LS. 2018 Desperate planktotrophs: decreased settlement selectivity with age in competent eastern oyster *Crassostrea virginica* larvae. Mar. Ecol. Prog. Ser. **599**, 93-106. (10.3354/meps12653)

[61] Cowan ZL, Dworjanyn SA, Caballes CF, Pratchett MS. 2016 Benthic predators influence microhabitat preferences and settlement success of crown-of-thorns starfish (*Acanthaste*r cf. *solaris*). Diversity **8**, 27. (10.3390/d8040027)

[62] Yamaguchi M. 1974 Growth of juvenile *Acanthaster planci* (L.) in the laboratory. Pac. Sci. **28**, 123-138.

[63] Sweatman H. 2008 No-take reserves protect coral reefs from predatory starfish. Curr. Biol. **18**, 598-599. (10.1016/j.cub.2008.05.033) 18644332

[64] Hock K, Wolff NH, Condie SA, Anthony KRN, Mumby PJ. 2014 Connectivity networks reveal the risks of crown-of-thorns starfish outbreaks on the Great Barrier Reef. J. Appl. Ecol. **51**, 1188-1196. (10.1111/1365-2664.12320)

[65] Wooldridge SA, Brodie JE. 2015 Environmental triggers for primary outbreaks of crown-of-thorns starfish on the Great Barrier Reef, Australia. Mar. Poll. Bull. **101**, 805-815. (10.1016/j.marpolbul.2015.08.049) 26460182

[66] Fabricius K, De'ath G. 2001 Environmental factors associated with the spatial distribution of crustose coralline algae on the Great Barrier Reef. Coral Reefs **19**, 303-309. (10.1007/s003380000120)

[67] Diaz-Pulido G, Cornwall C, Gartrell P, Hurd CL, Tran VD. 2016 Strategies of dissolved inorganic carbon use in macroalgae across a gradient of terrestrial influence: implications of the Great Barrier Reef in the context of ocean acidification. Coral Reefs **35**, 1327-1341. (10.1007/s00338-016-1481-5)

[68] Ringeltaube P, Harvey A. 2000 Non-geniculate coralline algae (Corallinales, Rhodophyta) on Heron Island, Great Barrier Reef (Australia). Bot. Mar. **43**, 431-454. (10.1515/BOT.2000.045)

[69] Doll PC, Uthicke S, Caballes CF, Diaz-Pulido G, Abdul Wahab MA, Lang BJ, Jeong SY, Pratchett MS. 2023 psbA and RbcL GenBank Accession numbers for coralline algae species identification. National Center for Biotechnology Information (NCBI). See https://www.ncbi.nlm.nih.gov/genbank/. 10.1098/rsbl.2022.0399 PMC9873471 36693424

[70] Doll PC, Uthicke S, Caballes CF, Diaz-Pulido G, Abdul Wahab MA, Lang BJ, Jeong SY, Pratchett MS. 2023 Data from: Settlement cue selectivity by larvae of the destructive crown-of-thorns starfish. *Dyrad Digital Repository*. (10.5061/dryad.gqnk98srv) PMC9873471 36693424

[71] Doll PC, Uthicke S, Caballes CF, Diaz-Pulido G, Abdul Wahab MA, Lang BJ, Jeong SY, Pratchett MS. 2023 Settlement cue selectivity by larvae of the destructive crown-of-thorns starfish. *Figshare*. (10.6084/m9.figshare.c.6384907) PMC9873471 36693424

